# Case Report: Malacoplakia Due to *E. coli* With *Cryptococcus albidus* Infection of a Transplanted Kidney in a Patient With Recurrent Urinary Tract Infection

**DOI:** 10.3389/fmed.2021.721145

**Published:** 2021-09-14

**Authors:** Ziyan Yan, Wenfeng Deng, Yuchen Wang, Yanna Liu, Hengbiao Sun, Renfei Xia, Wenli Zeng, Jian Geng, Gui Chen, Xiaolong He, Jian Xu, Chin-Lee Wu, Yun Miao

**Affiliations:** ^1^Department of Transplantation, Nanfang Hospital, Southern Medical University, Guangzhou, China; ^2^Department of Microbiology and Infectious Disease Center, School of Basic Medical Sciences, Peking University Health Science Center, Beijing, China; ^3^Department of Laboratory, The Third Affiliated Hospital of Southern Medical University, Guangzhou, China; ^4^Department of Pathology, Nanfang Hospital, Southern Medical University, Guangzhou, China; ^5^Guangdong Provincial Key Laboratory of New Drug Screening, School of Pharmaceutical Sciences, Southern Medical University, Guangzhou, China; ^6^Guangdong Provincial Key Laboratory of Tropical Disease Research, Department of Microbiology, School of Public Health, Southern Medical University, Guangzhou, China; ^7^Departments of Urology and Pathology, Harvard Medical School, Massachusetts General Hospital, Boston, MA, United States

**Keywords:** cryptococcoma, malacoplakia, *Cryptococcus albidu*s, *Escherichia coli 09-02E*, transplanted kidney, metagenome sequencing

## Abstract

**Background:** Colonization of *Cryptococcus* rarely occurs in a graft. This study reports a case of malacoplakia and cryptococcoma caused by *E. coli* and *Cryptococcus albidus* in a transplanted kidney, with detailed pathology and metagenome sequencing analysis.

**Case Presentation:** We presented a case of cryptococcoma and malacoplakia in the genitourinary system including the transplant kidney, bladder, prostate, and seminal vesicles caused by *Cryptococcus albidus* and *Escherichia coli* in a renal-transplant recipient. Metagenome sequencing was conducted on a series of samples obtained from the patient at three different time points, which we termed Phase I (at the diagnosis of cryptococcoma), Phase II (during perioperative period of graftectomy, 3 months after the diagnosis), and Phase III (2 months after graftectomy). Sequencing study in the Phase I detected two and four sequences of *C. albidus* respectively in cerebrospinal fluid (CSF) and feces, with resistant *Escherichia coli 09-02E* presented in urine and renal mass. A 3-month antibiotic treatment yielded a smaller bladder lesion but an enlarged allograft lesion, leading to a nephrectomy. In the Phase II, two sequences of *C. albidus* were detected in CSF, while the *E. coli 09-02E* continued as before. In the Phase III, the lesions were generally reduced, with one *C. albidus* sequence in feces only.

**Conclusions:** The existence and clearance of *Cryptococcus* sequences in CSF without central nervous system symptoms may be related to the distribution of infection foci *in vivo*, the microbial load, and the body's immunity. Overall, this study highlights the need for enhanced vigilance against uncommon types of *Cryptococcus* infections in immunocompromised populations and increased concern about the potential correlation between *E. coli* and *Cryptococcus* infections.

## Introduction

As an opportunistic pathogenic fungus, *Cryptococcus* is the third most common invasive fungi in solid organ transplantation (SOT) (1). The prevalence of cryptococcosis in this population is 0.2 to 5.8%, with a total mortality rate ranging from 20 to 50% (1, 2). Human immunodeficiency virus-infected patients and SOT recipients are at the highest risk for *Cryptococcus* infection (3), with *Cryptococcus neoformans* and *Cryptococcus gattii* the most common causes of cryptococcosis. Once inhaled, *Cryptococcus* can disseminate to the whole body or colonize in host tissue through the bloodstream, resulting in diseases such as cryptococcal meningoencephalitis, pulmonary cryptococcosis and cryptococcal granuloma (2, 4, 5). However, *Cryptococcus albidus* infection is rare, with skin the most commonly involved organ and a mortality rate of around 28% (5/18) (6).

Only one case of localized cryptococcal lesion in transplant kidney has been reported so far (7). Herein we reported the first case of pathology- and metagenome sequencing-proven cryptococcoma caused by *C. albidus* of a transplanted kidney in a patient presenting with urinary tract infection (UTI) of *Escherichia coli* and BK polyomavirus viruria. This study sheds light on the correlation between drug-resistant *E. coli* and *Cryptococcus* infection. The results suggest that alteration of the immune microenvironment caused by a long-term infection, such as *E. coli* infection, may be the key reason for the colonization of *Cryptococcus* in uncommon sites of the body, like an allograft.

## Case Description

### Clinical History

**Figure 1:**
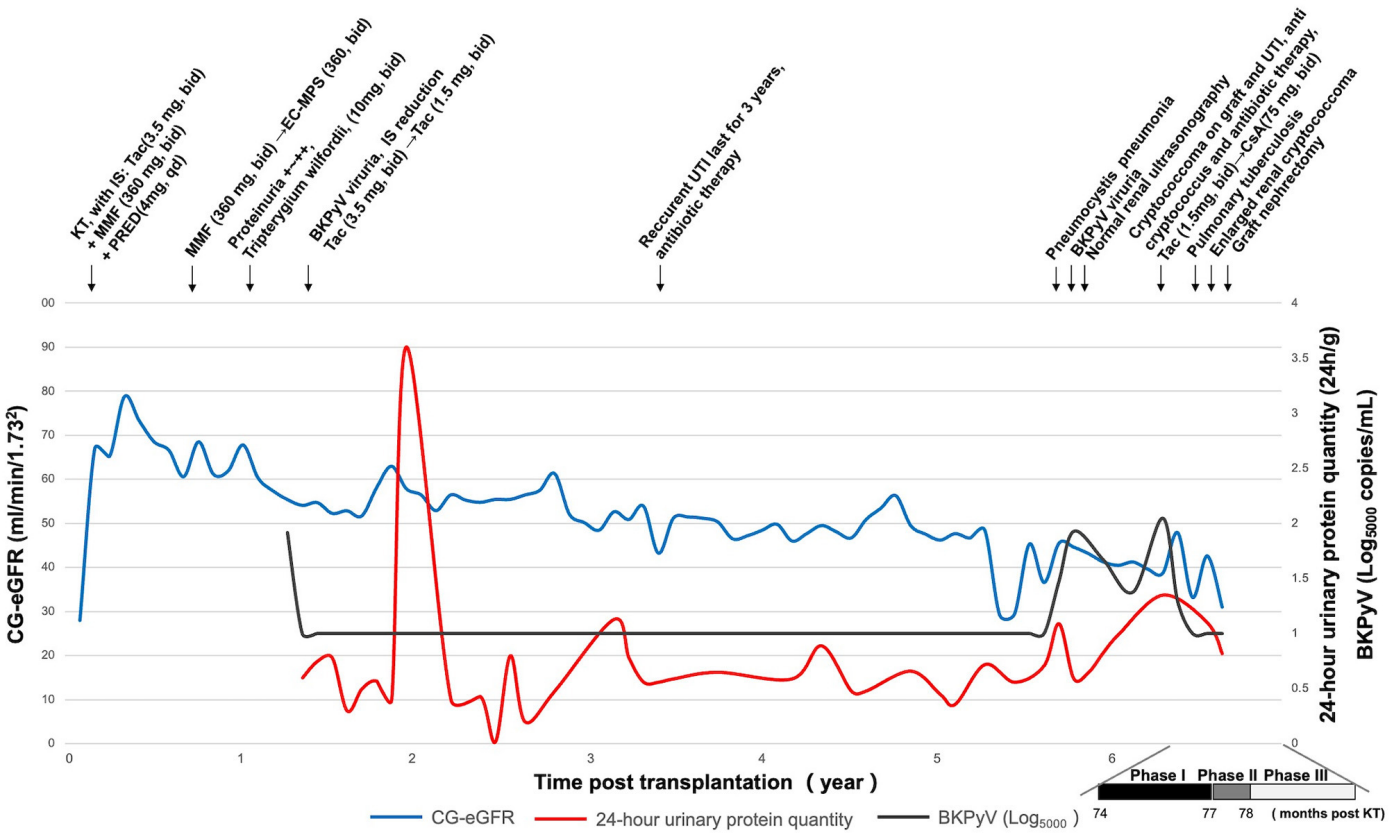
Clinical history of the patient. CG-eGFR, estimated glomerular filtration rate by the Cockcroft-Gault; KT, kidney transplantation; IS, immunosuppressant; Tac, tacrolimus; MMF, mycophenolate mofetil; PRED, prednisone; EC-MPS, mycophenolate sodium enteric-coated tablets; CsA, cyclosporine; BKPyV, BK polyomavirus; UTI, urinary tract infection.

A 50-year-old male with end-stage renal disease received a left kidney transplant from a deceased male donor who died in a motor vehicle accident in March 2013. After renal transplantation, the patient received a maintenance immunosuppressive regimen consisted of tacrolimus (3.5 mg, bid), mycophenolate mofetil (360 mg, bid), and prednisone (4 mg, qd). At 11 months postoperatively, 1+ to 2+ proteinuria was found on a routine urine examination. The proteinuria was relieved after treatment with Tripterygium glycosides tablets (10 mg, bid). At 15 months postoperatively, the patient developed BKV viruria with a urinary viral load of 1.25 × 10^7^ copies/mL (normal range for reference, <5,000 copies/mL). The viral load was undetectable after the dosage of tacrolimus was reduced to 1.5 mg BID and treating with immunoglobulin (infusion). In April 2016, the patient had a chronic rejection reaction and the 24-h urinary protein quantity increased to 1.13 g/24 h. To maintain the allograft function and suppress proteinuria, the corresponding treatment regimen was methylprednisolone (40 mg) combined with cyclophosphamide (0.2 g) intravenous drip for 3 days/month. After three courses of treatment, the 24-h urinary protein quantity decreased to 0.56 g/24 h. The patient has had recurrent symptoms of UTI such as frequent and urgent urination without obvious inducement since June 2016. Regular outpatient review of urinary examination revealed leukocytes fluctuating from 1+ to 3+. *E. coli* was detected in the midstream urine culture and intravenous cefoperazone sodium sulbactam (1.5 g, 1/12 h) was given for 1 week. In September 2018, the patients came to the hospital because of cough for 1 day. mNGS of the alveolar lavage fluid indicated pneumosporidiosis and blood tested positive for herpes simplex virus. The pulmonary infection resolved after treated with compound sulfamethoxazole tablets (480 mg, bid). Besides, serum creatinine decreased from 305 μmol/L to 245 μmol/L. However, BKV viruria relapsed with the urinary viral load fluctuated from 2.84 × 10^5^ copies/mL to 3.81 × 10^7^ copies/mL. The immunosuppressive regimen was adjusted to tacrolimus (1.5 mg, bid), mycophenolate sodium enteric-coated tablets (180 mg, bid), and prednisone (4 mg, qd). In November 2018, the patient's serum creatinine was 197 μmol/L, and color Doppler examination of the transplanted kidney and renal vessels showed no significant abnormalities. Regular color Doppler ultrasound examinations of the allograft and transplanted kidney vessels were performed every 6 months after transplantation, all showing neither significant abnormalities nor transplanted kidney masses until the current admission. The patient had not undergone an allograft puncture biopsy within 3 years after renal transplantation. His postoperative serum creatinine level was 190 μmol/L. And the postoperative glomerular filtrate rate (GFR) was summarized in Figure 1. The patient was admitted to hospital in May 2019 because of frequent and urgent urination. The clinical course of the patient is summarized in three phases according to the disease progression.

#### Phase I

**Figure 2:**
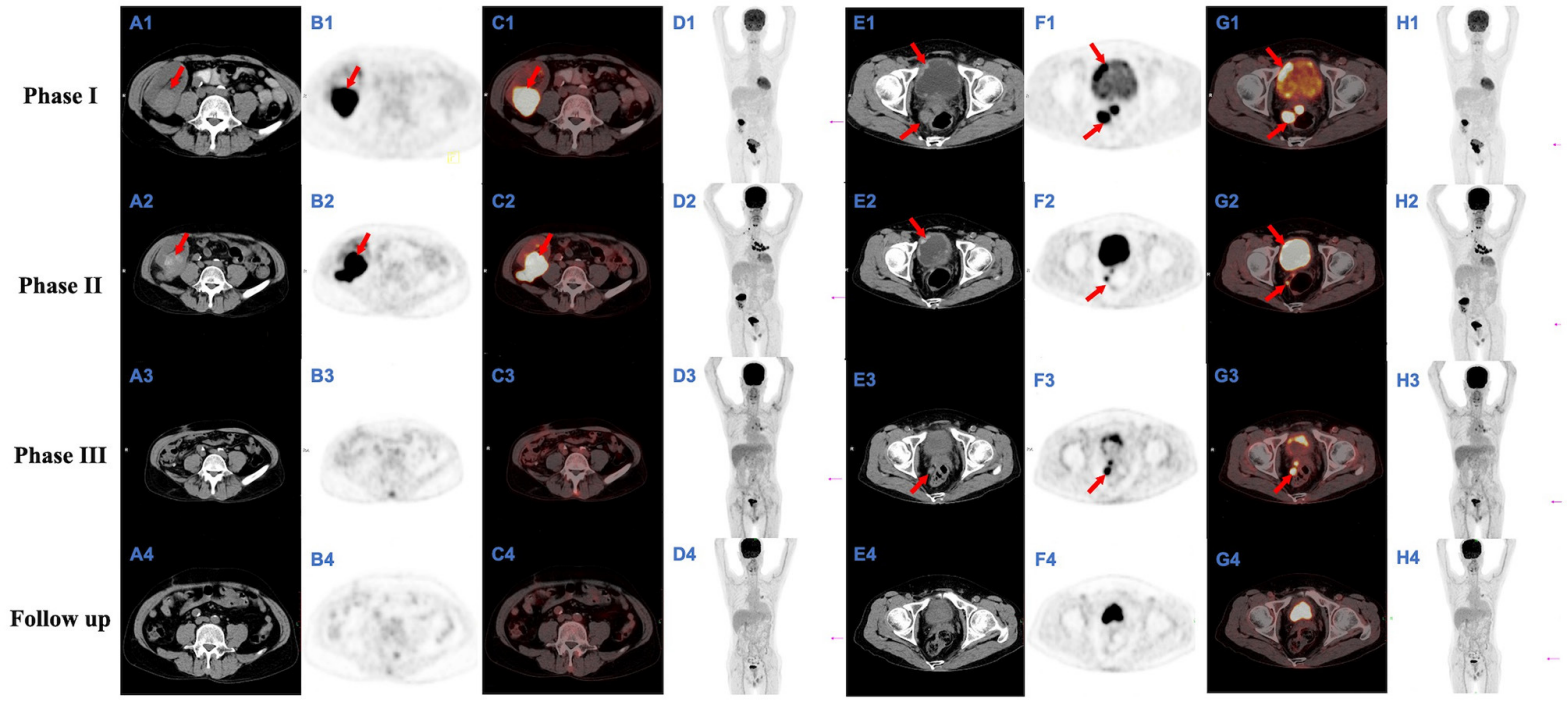
FDG-FDG-PET/CT images. Full-body PET-maximum intensity projection (MIP) **(D1–4, H1–4)** show several space-occupying hypermetabolic lesions; they also indicate the positions (magenta arrows) of the slice shown (**A1–4, B1–4, C1–4**, and **E1–4, F1–4, G1–4**, respectively); an axial CT image (**A1–4** and **E1–4**, respectively), axial PET image (**B1–4** and **F1–4**, respectively), and axial FDG-PET/CT fusion image (**C1–4** and **G1–4**, respectively) from a single plane show a mass of hypermetabolic lesions in the transplanted kidney and bladder respectively; the largest of which (**A1–2**, **B1–2**, and **C1–2**, red arrows), ~4.3 × 4.9 × 4.1 cm and 6.0 × 4.0 × 4.7 cm in size, with maximal standardized uptake value (SUVmax) of 20.3 and 31.0 and average standardized uptake value (SUVave) of 8.8 and 17.4 respectively; the largest of which (**E1–3**, **F1–3**, and **G1–3**, red arrows), ~4.7 × 4.1 × 5.5 cm, 3.8 × 2.3 × 3.6 cm, and 2.8 × 1.4 × 2.5 cm in size, with SUVmax of 21.9, 21.4, and 6.4 and SUVave of 8.7, 8.9, and 4.3 respectively.

**Figure 3:**
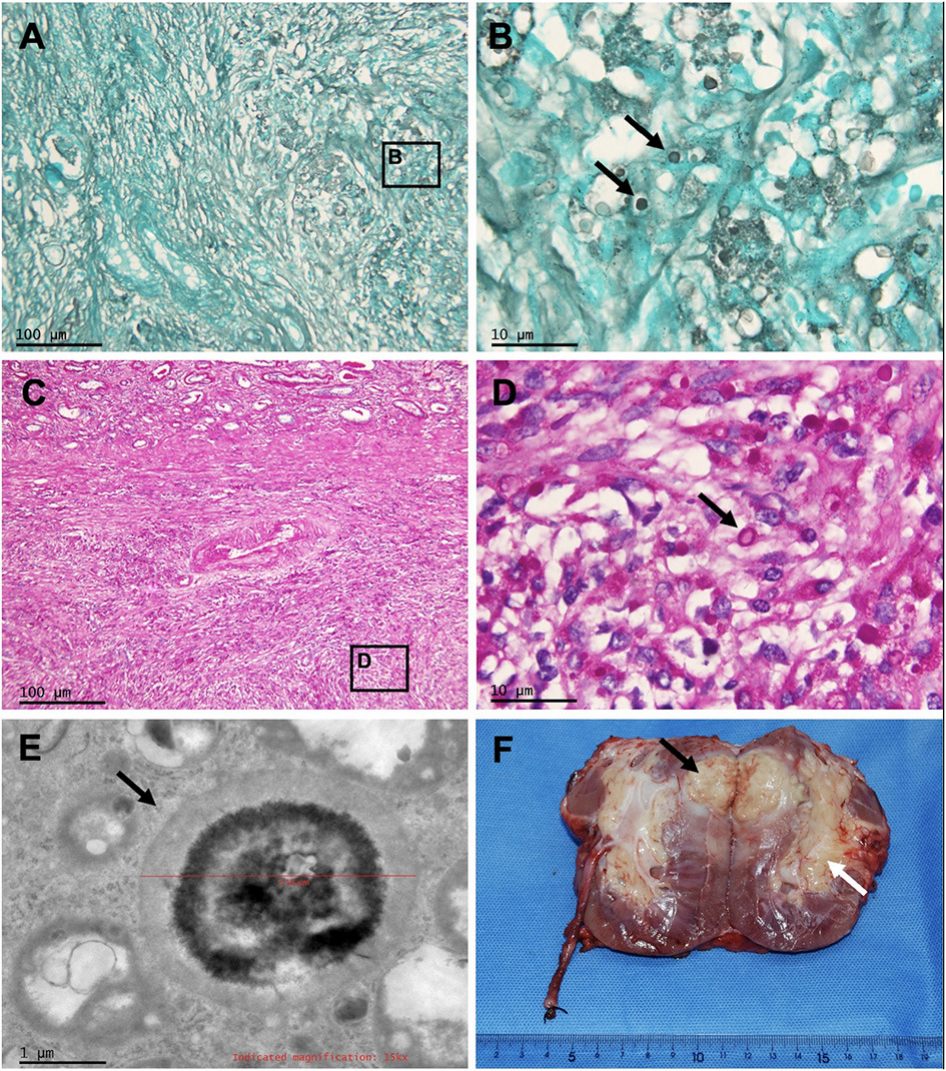
Pathology of the masses of the transplanted kidney, and gross photograph of the allograft nephrectomy specimen. Periodic acid-Schiff staining and Grocott methenamine silver staining of renal mass at 100 × **(A,C)** and at 1,000 × **(B,D)** show granuloma caused by Cryptococcus (arrows). Electron microscopy of renal mass **(E)** shows the Cryptococcus (arrow). **(F)** Renal cryptococcoma (black arrow) and enlarged renal crptococcoma (white arrow).

In May 2019, the patient was admitted to hospital for recurrent frequent and urgent urination. On admission, his serum creatinine level was 229 μmol/L. Ultrasound and PET/CT showed solid space-occupying lesions in the upper pole of the transplanted kidney and bladder, prostate and seminal vesicles (Figures 2A1–H1). Biopsy of the graft kidney and bladder lesions revealed cryptococcal granulomas, with cystoscopy results provided in Supplementary Figure 1. Extended-spectrum β-lactamase positive *E. coli* was cultured from both renal graft tissue and midstream urine. To rule out systemic cryptococcosis, a lumbar puncture was performed to collect CSF and CSF opening pressure measured. Cell counts and biochemical parameters in CSF, and CSF opening pressure were normal. Ink staining and *Cryptococcus* antigen detection of CSF were both negative. No significant abnormalities were observed in brain MRI and chest CT scans. Based on the imaging and pathological findings, the patient was diagnosed with cryptococcoma and malacoplakia of the genitourinary system including transplanted kidney, bladder, prostate and seminal vesicles (Figures 2A1–H1 and Figure 3), accompanied by an UTI of *E. coli*. The patient was treated with meropenem (0.5 g twice a day), and fluconazole (50 mg twice a day) combined with flucytosine (0.5 g twice a day). Meanwhile, immunosuppression was reduced by conversion from tacrolimus (1.5 mg twice a day) to cyclosporine (100 mg twice a day). Additionally, cyclosporine was later adjusted to 75 mg BID after use of the antifungal drug, fluconazole, which can affect the concentration of cyclosporine. The concentrations of immunosuppressants from renal transplantation to transplant nephrectomy were shown in Supplementary Figure 4. After 10 days' treatment, his renal function improved and the serum creatinine decreased from 229 to 185 μmol/L. In July 2019, his chest X-ray showed a soft tissue shadow in the left hilum, which was diagnosed as pulmonary tuberculosis by bronchoscopy; and the patient was thus treated with isoniazid (300 mg, once daily), ethambutol (750 mg, once daily) and levofloxacin (250 mg, once daily).

#### Phase II

Follow-up ultrasound and FDG-PET/CT examination in August 2019 showed that although the area of bladder lesion was significantly reduced from 4.7 × 4.1 × 5.5 cm to 3.8 × 2.3 × 3.6 cm, the lesion in the transplanted kidney was enlarged from 4.3 × 4.9 × 4.1 cm to 6.0 × 4.0 × 4.7 cm, with the cryptococcoma and malacoplakia in the upper pole of the transplanted kidney protruding into the adjacent Gerota's fascia (Figures 2A2–H2). Fluconazole was given at 100 mg BID against cryptococcal infection while the anti-Escherichia coli as well as the anti-tuberculosis regimen was maintained, as described in Phase I. The net immunosuppression status of an individual is influenced by the immunosuppression regimen and individual susceptibility and can be assessed by immunosuppressive drug concentrations, peripheral blood leukocyte counts, lymphocyte counts, and viral infection conditions. The peripheral blood leukocyte and lymphocyte counts of this case were lower compared to the average level during uninfected period (Supplementary Figure 5). The presence of pneumosporidiosis, herpes simplex virusemia, and BKV uremia were all suggestive of a low net immunosuppressive status. To stop the disease progression, the patient received a transplant nephrectomy 1 week later.

#### Phase III

After transplant nephrectomy, the patient's immunosuppressants were discontinued. He was on dialysis three times a week. The anti-tuberculosis treatment was changed to isoniazid (300 mg, once daily), ethambutol (870 mg, once every 2 days) and levofloxacin (250 mg, once every 2 days), while the anti-*Cryptococcus* treatment was changed to fluconazole (100 mg, twice a day) and flucytosine (0.5 g, three times a day). After 2 months, a follow-up FDG-PET/CT showed that the lesions in the urogenital system and lung were significantly reduced compared with the last examination (Figures 2A3–H3). Six months post-transplant nephrectomy, the lesions in the genitourinary system were eventually eliminated (Figures 2A4–H4).

### Pathology

Gross inspection of the transplanted kidney showed that the resected transplant volume was 11 × 9.0 × 5.0 cm with its capsule closely adherent to the surrounding fat. A solid yellow mass of 6.0 × 4.0 × 4.7 cm was found in the upper pole renal parenchyma without breaking through the renal capsule. Another 5.0 × 2.5 × 2.5 cm yellow mass was found in the hilar sinus fat (Figure 3F). The pathological changes of the transplanted kidney lesions were consistent with *Cryptococcus* infection (Figures 3A–E).

### Metagenome Sequencing

Metagenome sequencing revealed that only two and four sequences of *C. albidus* were respectively detected in the CSF and feces specimens in Phase I. Two sequences of *C. albidus* were detected in the CSF specimen in Phase II. After transplantation nephrectomy, withdrawal of immunosuppressants and anticryptococcal therapy for 2 months, one sequence of *C. albidus* was detected in the feces specimen but no sequence in CSF in Phase III. All sequences were typed as *[Cryptococcus] albidus var. albidus strain NRRL Y-1402* (Table 1). The type of the *E. coli* detected in urine and granuloma of the allograft in Phase I and Phase II was *E. coli 09-02E* (Table 1).

**Table 1:** Metagenome sequencing results of Cryptococcus and *Escherichia coli* in patient' specimens.

**Phase**	**Specimen**	***Cryptococcus* (Sequence number, coverage)**	***Escherichia coli* (Sequence number, coverage)**
I	Blood	—^*^	—
	CSF	*[Cryptococcus] albidus var. albidus strain NRRL Y-1402* (2, 100.00%)	—
	Feces	*[Cryptococcus] albidus var. albidus strain NRRL Y-1402* (4, 100.00%)	*Escherichia coli strain AR_0006* (357,907, 100.00%)
	Urine	—	*Escherichia coli 09-02E* (110,960, 100%)
	Allograft tissue	—	*Escherichia coli 09-02E* (14,751, 100%)
	Blood	—	—
II	CSF	*[Cryptococcus] albidus var. albidus strain NRRL Y-1402* (2, 100.00%)	—
	Feces	—	*Escherichia coli strain 2012C-4221*n (786, 100%)
	Urine	—	*Escherichia coli 09-02E* (32,995, 100%)
	Sputum	—	—
	Allograft tissue (uninvolved)	—	—
	Allograft tissue (previous lesion)	—	—
	Allograft tissue (new-born lesion)	—	*Escherichia coli 09-02E* (12,361, 100%)
III	Blood	—	—
	CSF	—	—
	Feces	*[Cryptococcus] albidus var. albidus strain NRRL Y-1402* (1, 95.33%)	*Escherichia coli strain Ec-2Lar* (69,700, 100%)
	Sputum	—	—

* *Undetected or not consistent with the filter criteria*.

### Culture *in vitro*

In order to understand the interactions between *E. coli* and *Cryptococcus*, we cocultured the two microorganisms *in vivo*. After an 8-h co-culture of *E. coli 09-02E* filtrate and *Cryptococcus neoformans JEC21* (ATCC@96910) *in vitro*, the *Cryptococcus* counts in the control group (without *E. coli* filtrate) and the experimental group (adding 80, 160, 320, 640, and 1,280 μl *E. coli* filtrate, respectively) were respectively 2.46 ± 0.52 × 10^5^/mL, 2.30 ± 0.57 × 10^5^/mL, 2.83 ± 0.72 × 10^5^/mL, 3.13 ± 0.76 × 10^5^/mL, 3.09 ± 0.61 × 10^5^/mL, and 2.60 ± 0.63 × 10^5^/mL. Comparison between the control group and the experimental Group III (320 μl *E. coli* filtrate) and Group IV (640 μl *E. coli* filtrate) showed a statistically significant difference (*P* < 0.05) (Supplementary Figure 2), indicating that *E. coli* at these concentrations may stimulate cryptococcal growth.

## Discussion

This is the first sequencing study, to our knowledge, of malacoplakia and cryptococcoma of *E. coli* and *C. albidus* in the transplanted kidney. *Cryptococcus* usually attacks the immunocompromised population, resulting in mostly systemic infection (8). Its colonization in the transplanted kidney is extremely rare, with only one case reported before (7). In this case, a 50-year-old male patient with a recurrent UTI of *E. coli* for 3 years developed cryptococcoma in the transplanted kidney on the 74th month after transplantation. The granuloma disseminated to urogenital organs such as the bladder, prostate, and seminal vesicle. Metagenome sequencing identified the *[Cryptococcus] albidus var. albidus strain NRRL Y-1402* as the culprit. And the drug-resistant *E. coli 09-02E*, first detected in feces from healthy Vietnamese people in 2018, and with unclear pathogenic mechanism and unique urinary system properties (9), arose after the long-term antibiotic use presumably due to selective pressure. Microbiological analysis of kidney transplant preservation fluids was performed prior to transplantation and showed negative results. The patient's recurrent UTI of *E. coli* began 3 years postoperatively, so the *E. coli* infection was considered non-donor-derived in this case.

Although co-infection of *Cryptococcus* and *E. coli* in the same lesion are rarely detected or reported, we speculated that there is an inevitable relationship between them: long-term repeated antibiotics use leads to dysregulation of bacterial flora drug-resistant strains (10), and promotes the emergence of drug-resistant *E. coli* and *Cryptococcus* colonization. In this case, *E. coli* might have invaded the transplanted kidney prior to *Cryptococcus*.

Pathological examination revealed granuloma and focal inflammatory cell infiltration and fibrosis in the interstitium. Microbiological culture and metogenomic sequencing results of the transplanted kidney tissues both showed a large number of *E. coli*. The α-hemolysase released by *E. coli* can cause renal injury and cicatrization, facilitate the formation of abscesses or granulomas, and block urine excretion in the collecting duct (11), which may be the biological causes of *Cryptococcus* retention. Studies have shown that *E. coli* infection alters the immune microenvironment of the infected foci, such as the inflammatory response induced by the activation of cytokines TNF-α, IL-1, IL-6, and IL-8 (11, 12). This immune microenvironment may be the fertile soil for *Cryptococcus* infection and colonization in *E. coli* infectious foci.

Interactions between fungi and bacteria are common (13). Our *in vitro* co-culture result showed that metabolites of *E. coli* at certain concentration may stimulate cryptococcal growth, suggesting correlated growth between *E. coli* and *Cryptococcus*. Urinary susceptible *E. coli* is an important co-factor of multiple stress factors involved in the generation of melanin which is a necessary pathogenic factor for *Cryptococcus*, assisting in the removal of oxygen free radicals and averting the onset of oxidative stress response (14). In UTI, the genes involved in the Cu^1+^ efflox system of *E. coli* are highly up-regulated, and the copper efflox of *E. coli* may be the source of the copper intake of *Cryptococcus* (15), which may facilitate the infection and colonization of *Cryptococcus*.

*Cryptococcus* infection within 30 days after transplantation is generally considered donor-derived, and the median time for non-donor-derived *Cryptococcus* infection is 16–21 months after transplantation (2, 16, 17). Combined with the fact that the patient had no history of pathogen exposure, we suspected that the *Cryptococcus* spores were accidentally inhaled into the lung rather than donor-derived *Cryptococcus* infection, traversing pulmonary capillaries into peripheral blood circulation for systemic dissemination. The *E. coli* infection brought about changes in the transplanted kidney's immune microenvironment that promoted *Cryptococcus* which is used to manifest transient or latent infection to spread through the blood, to colonize in the transplanted kidney and gradually expand its range. After that, *Cryptococcus* proceeded down the urinary tract to the bladder or even prostate duct. At the same time, *Cryptococcus* can breach the blood-brain barrier and enter the CSF. The possible routes of infection were shown in Supplementary Figure 3. However, since pathogenicity was related to *Cryptococcus* infection foci *in vivo*, the fungal load *in situ* and the body's immunity, sequences of *C. albidus* could be detected in CSF even in an absence of cryptococcal meningoencephalitis symptoms.

The inconsistent outcomes of the lesions in bladder and transplanted kidney may resulted from that the granuloma created barriers around the infection site to prevent drugs entering the renal lesions. In contrast, the bladder lesion was relatively superficial and had long-term exposure to running urine that contained antifungal metabolites. The fluconazole and flucytosine taken by this patient were metabolized by the kidney and excreted from the urine, so the therapeutic effect toward the bladder lesion was significantly superior to that of the transplanted kidney.

Currently, the detection technology of *Cryptococcus* antigens is based on *C. neoformans* and *C. gattii*. The sensitivity of *C. albidus* detection rate is 75% lower than that of *C. neoformans* and *C. gattii*, leading to the false-negative error in preliminary clinical screening (18). This may have contributed to the paradox between pathology-proven cryptococcoma and negative *Cryptococcus* latex antigen test of CSF and the culture of the blood, urine, CSF, sputum, feces and renal graft tissues in our study. Metagenome sequencing can act as an effective technical complement to pathogen detection in transplant recipients.

## Conclusions

In conclusion, this study reported the first sequencing study of cryptococcoma and malacoplakia formed by *C. albidus* and *E. coli* in a transplanted kidney. This case suggests a possible synergistic relationship between *Cryptococcus* colonization and drug-resistant *E. coli* infection in the transplanted kidney. At the same time, we should be alert to the infection caused by rare *Cryptococcus* in clinical practice. In addition to traditional diagnostic methods such as culture and immunoassay, metagenome sequencing can be utilized as an auxiliary diagnostic tool.

## Data Availability Statement

The datasets presented in this study can be found in online repositories. The names of the repository/repositories and accession number(s) can be found at: https://www.ncbi.nlm.nih.gov/, PRJNA719067.

## Ethics Statement

This study was approved by Nanfang Hospital Ethical Committee (NFEC-2020-044). Written informed consent was obtained from the patient for the publication of this case report.

## Author Contributions

YM, ZY, and WD participated in research design. ZY, YW, and YL participated in the writing of the paper. C-LW, JX, and YM performed critical revision of the manuscript for important intellectual content. ZY, HS, and GC participated in the performance of the research. YM took charge for obtaining funding. XH and JG performed administrative, technical, or material support. RX and WZ performed statistical analysis. YM and C-LW supervised the study. All authors read and approved the final version.

## Funding

This study was funded by National Natural Science Foundation of China (Gant No. 82070770), Natural Science Foundation of Guangdong Province (Grant No. 2020A1515010674), the Science and Technology Planning Project of Guangzhou (Grant No. 201803010109), the President Funding of Nanfang Hospital (Grant No. 2018B009, 2018C003), and College Students' Innovative Entrepreneurial Training Plan Program (Grant Nos. X202012121239, 202012121046).

## Conflict of Interest

The authors declare that the research was conducted in the absence of any commercial or financial relationships that could be construed as a potential conflict of interest.

## Publisher's Note

All claims expressed in this article are solely those of the authors and do not necessarily represent those of their affiliated organizations, or those of the publisher, the editors and the reviewers. Any product that may be evaluated in this article, or claim that may be made by its manufacturer, is not guaranteed or endorsed by the publisher.

## Supplementary Material

Supplementary Figure 1 Cystoscopy in Phase I. Pale round-shaped mucosal protrusions on the right side of the bladder, with 3 cm in diameter.

Supplementary Figure 2 Cryptococcus counts after 8 h co-culture of Escherichia coli filtrate *in vitro*. No *E. coli* filtrate was added to the control group (group I), and 80 (group II), 160 (group III), 320 (group IV), 640 (group V) and 1,280 (group VI) μl *E. coli* filtrate was added to the solution of Cryptococcus respectively. ^*^*P* < 0.05.

Supplementary Figure 3 Possible infection pathway of Cryptococcus () and *Escherichia coli* ().

Supplementary Figure 4 Dosages and whole blood trough levels of immunosuppressive regimens. MPA, mycophenolic acid.

Supplementary Figure 5 White blood cell counts and lymphocyte counts in peripheral blood of the patient. WBC, white blood cell; LYM, lymphocyte.
