# Supplementary figures and images for: Case Report: Malacoplakia Due to E. coli With Cryptococcus albidus Infection of a Transplanted Kidney in a Patient With Recurrent Urinary Tract Infection
Source: Front Med (Lausanne). 2021 Sep 14;8:721145. doi: 10.3389/fmed.2021.721145 (PMC8476786; doi:10.3389/fmed.2021.721145)

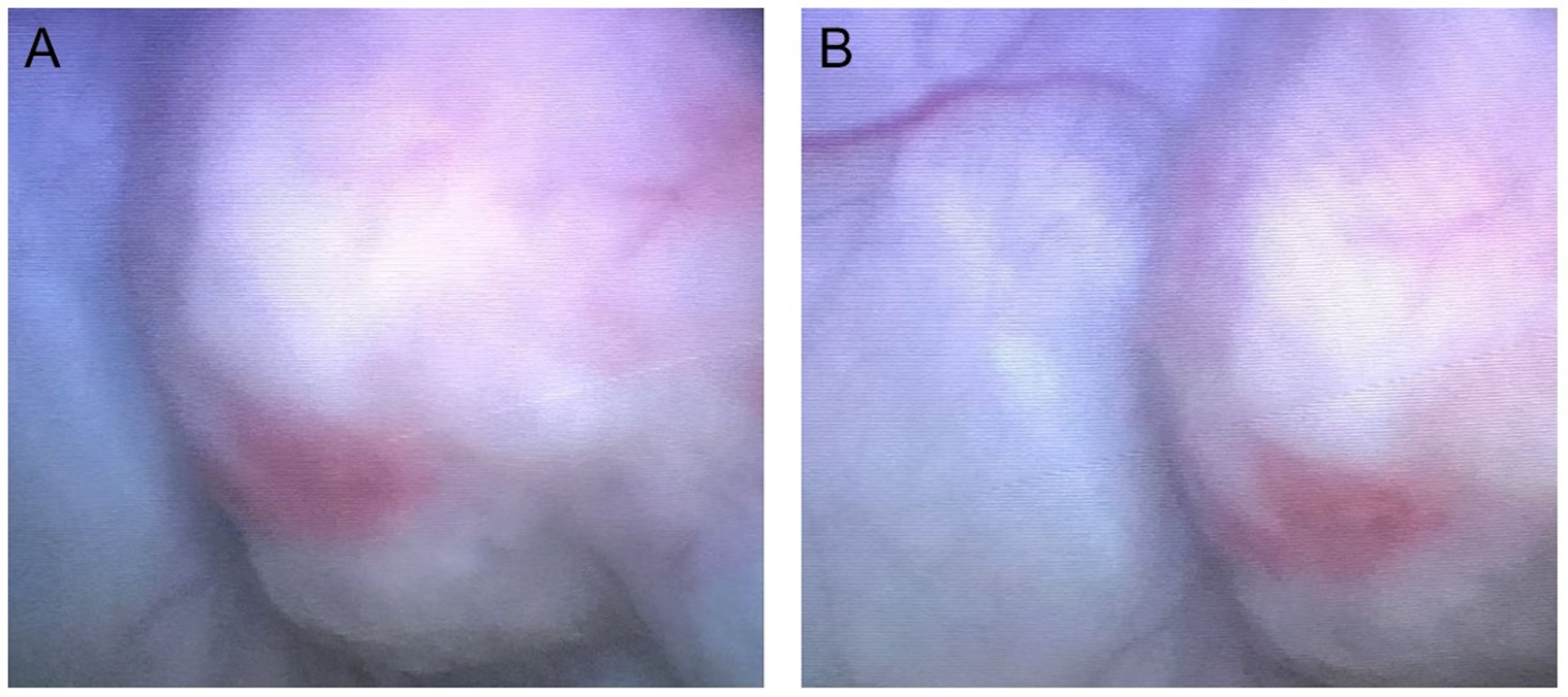

Supplement: Supplementary Figure 1 — Cystoscopy in Phase I. Pale round-shaped mucosal protrusions on the right side of the bladder, with 3 cm in diameter. [file Image_1.TIFF]

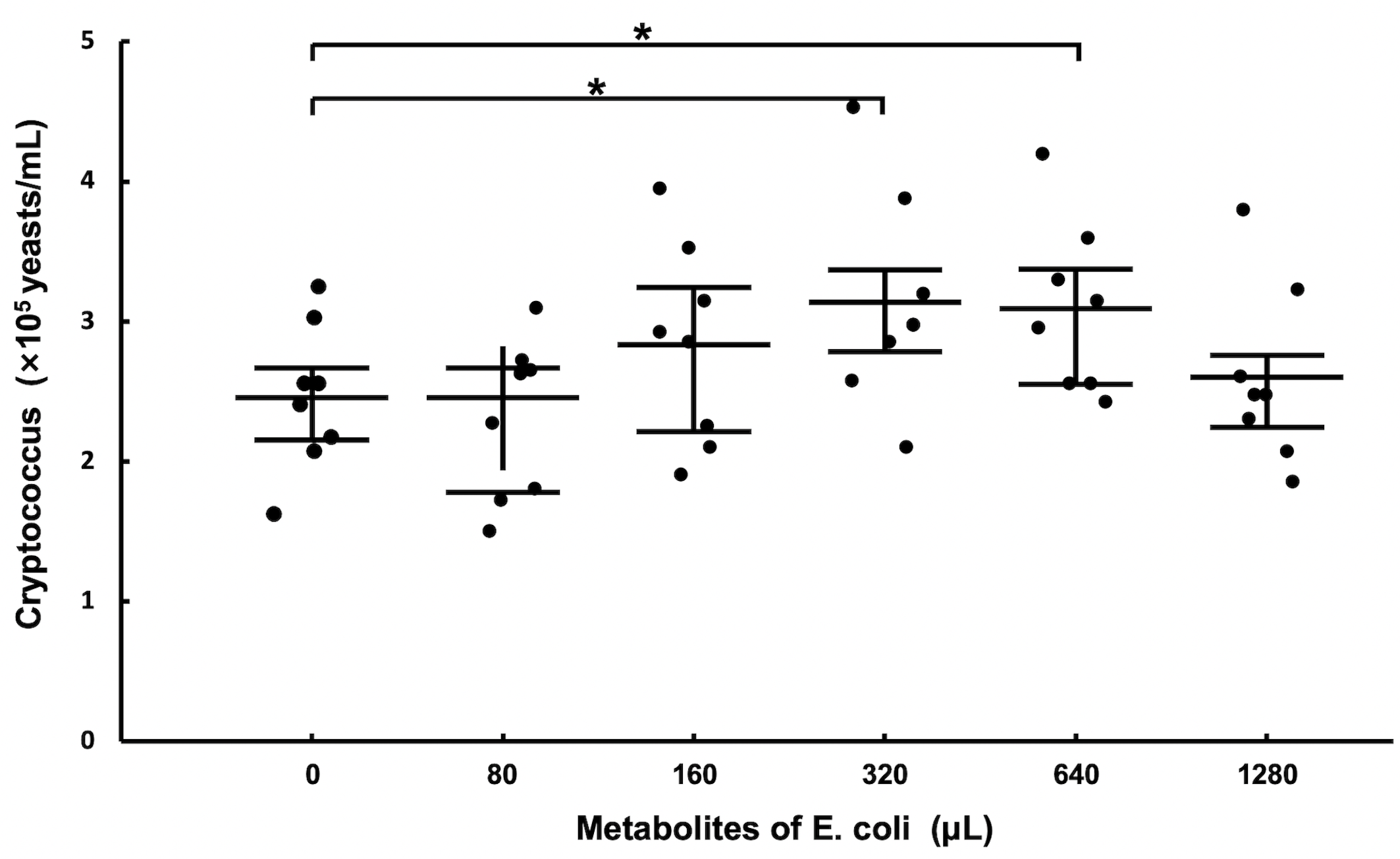

Supplement: Supplementary Figure 2 — Cryptococcus counts after 8 h co-culture of Escherichia coli filtrate in vitro. No E. coli filtrate was added to the control group (group I), and 80 (group II), 160 (group III), 320 (group IV), 640 (group V) and 1,280 (group VI) μl E. coli filtrate was added to the solution of Cryptococcus respectively. *P < 0.05. [file Image_2.TIFF]

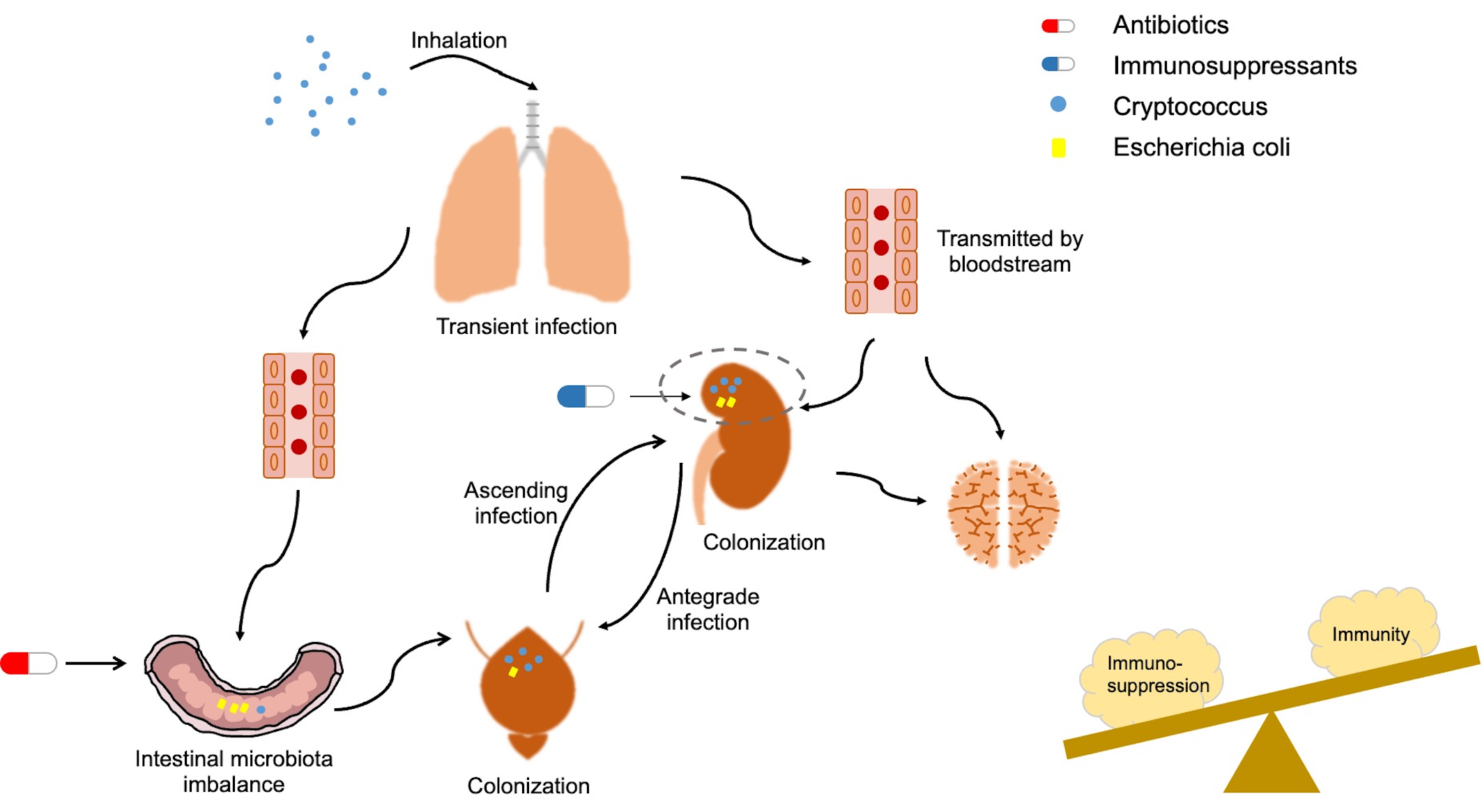

Supplement: Supplementary Figure 3 — Possible infection pathway of Cryptococcus () and Escherichia coli (). [file Image_3.TIFF]

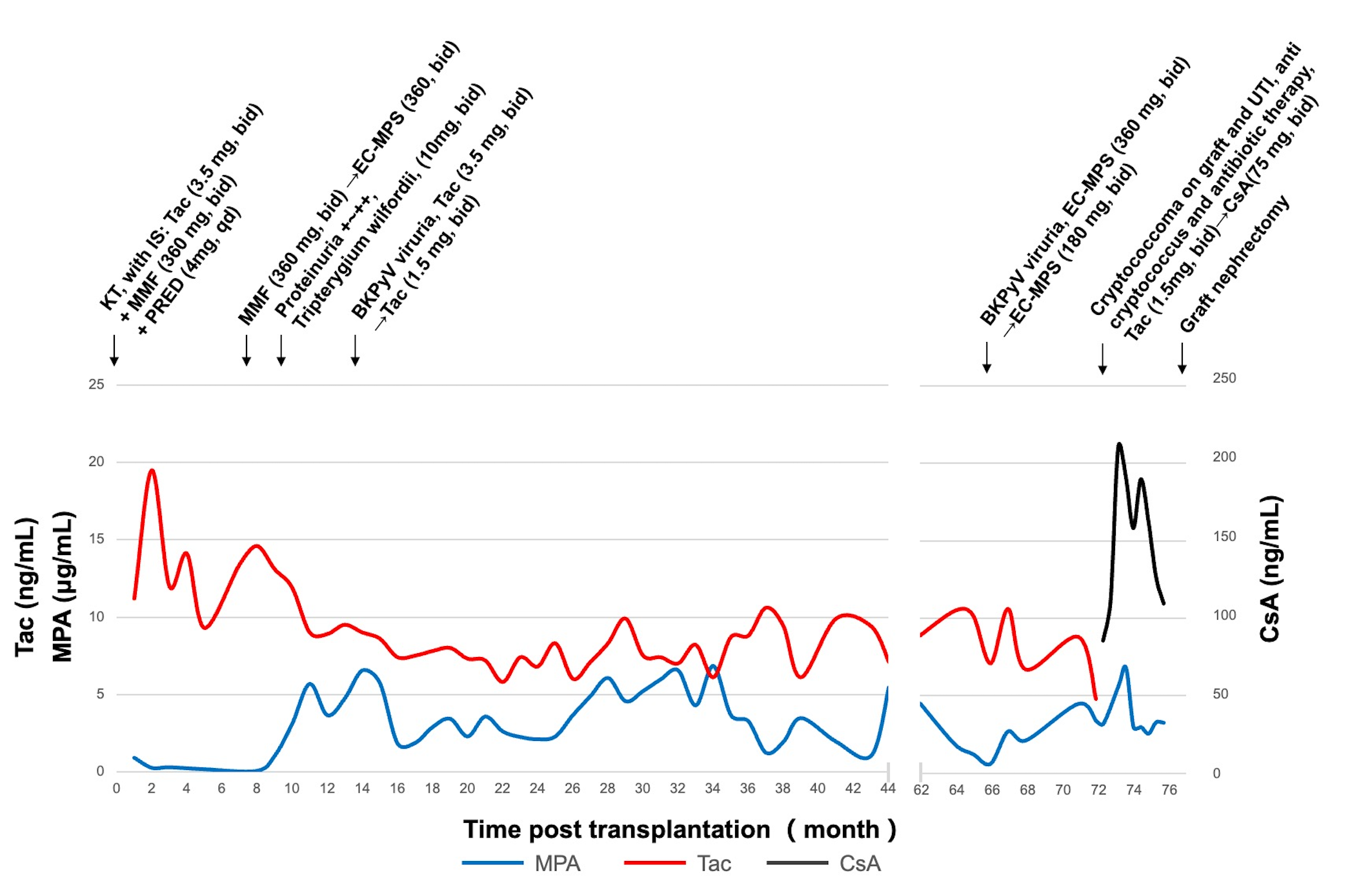

Supplement: Supplementary Figure 4 — Dosages and whole blood trough levels of immunosuppressive regimens. MPA, mycophenolic acid. [file Image_4.TIFF]

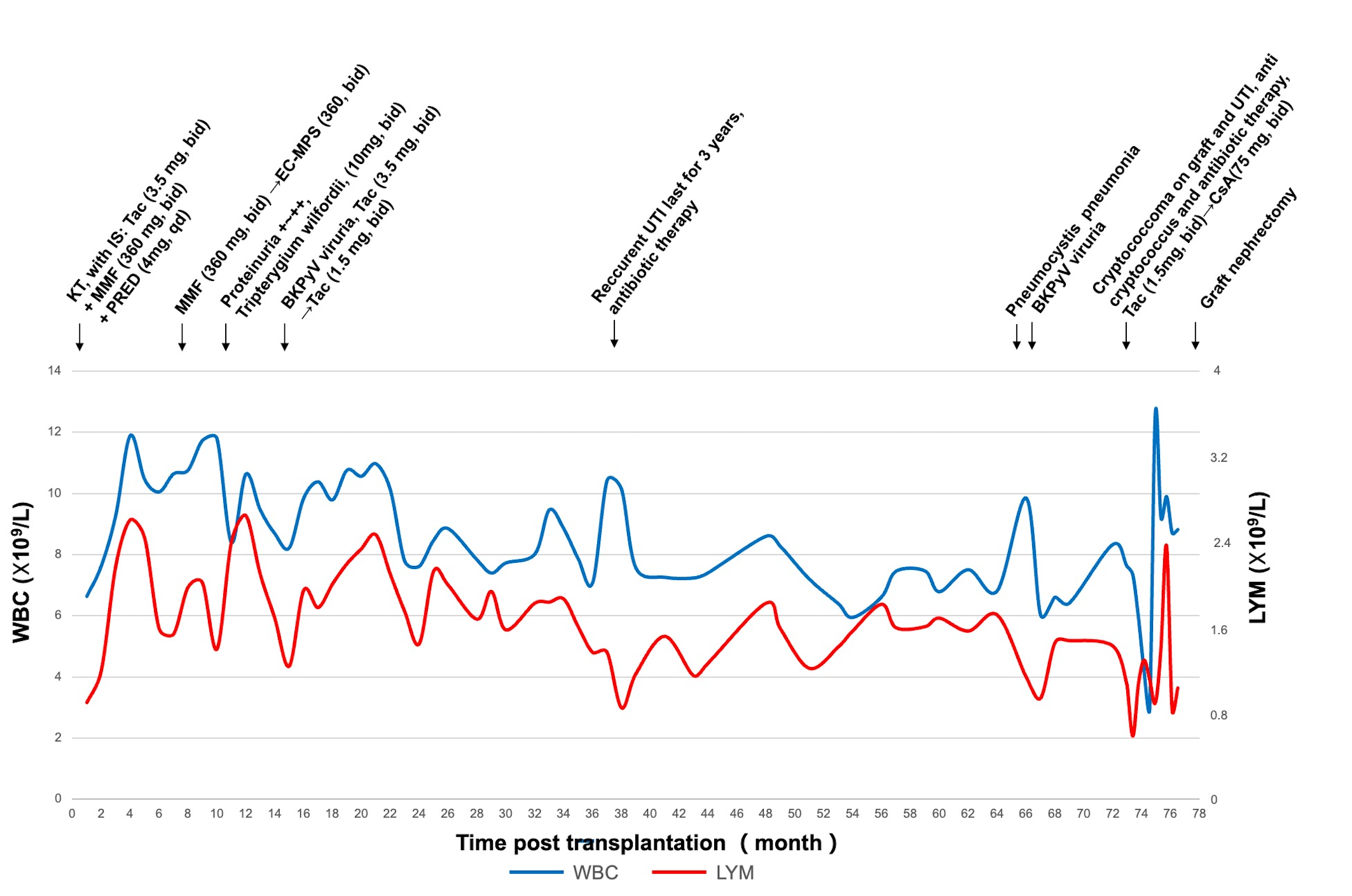

Supplement: Supplementary Figure 5 — White blood cell counts and lymphocyte counts in peripheral blood of the patient. WBC, white blood cell; LYM, lymphocyte. [file Image_5.TIFF]
